# The relationship between problem-solving ability and fatigue severity in people with multiple sclerosis

**Published:** 2020-01-05

**Authors:** Seyed Alireza Derakhshanrad, Emily Piven

**Affiliations:** 1 Department of Occupational Therapy, School of Rehabilitation Sciences, Shiraz University of Medical Sciences, Shiraz, Iran; 2 Rehabilitation Sciences Research Center, Shiraz University of Medical Sciences, Shiraz, Iran; 3 University of St. Augustine for Health Sciences, St. Augustine, Florida, United States of America

**Keywords:** Cognition, Fatigue, Multiple Sclerosis, Problem Solving

## Abstract

**Background:** The literature speculates that there may be a relationship between cognitive capacities and levels of fatigue in people with multiple sclerosis (MS), which has been under reported. This study has investigated one aspect of cognition by evaluating the association between problem-solving ability and the severity of fatigue.

**Methods:** A cross-sectional, descriptive study was used to investigate the association between levels of problem-solving ability and degrees of fatigue severity. Eighty-five participants living in the city of Shiraz, Iran, completed Cassidy Problem-Solving Inventory (PSI) and the Fatigue Severity Scale (FSS). Pearson’s Product Moment Correlation Coefficient was used to analyze the data.

**Results:** Problem-solving was inversely associated with fatigue (r = -0.381, P = 0.009), whereby higher levels of problem-solving ability were associated with lower degrees of fatigue.

**Conclusion:** The results of this study contributed to the ongoing debate about the linkage between cognition and fatigue in people with MS, suggesting an association between problem-solving ability and fatigue severity. A possible implication would be the importance of problem-solving training for people with MS.

## Introduction

Multiple sclerosis (MS) is an inflammatory demyelinating disease of the central nervous system characterized by sensory and motor impairment as well as fatigue and cognitive difficulties.^1^ Fatigue, a highly subjective feeling that may contribute to declines in cognitive and motor function, is acknowledged to be one of the most disabling symptoms among people with MS.^2^ Besides, people with MS experience dysfunction in various aspects of cognitive domains including attention, information processing, executive function, and memory.^3^ Executive function deficit is one of the most widely reported cognitive impairments in MS, leading mainly to difficulties in cognitive abilities such as planning, problem-solving, and self-monitoring.^4^ Of particular, interest to this study is the cognitive ability of problem-solving that is considered to be a coping strategy requiring foresight, planning, and development of perspective in people with MS.^5^

Whilst recent development in the field of MS proposes the theory of cognition-fatigue linkage,^6^ there is still uncertainty about which aspect of the cognitive function is more salient, emphasizing the need for more research on investigating such a linkage.^6^

Consequently, the objective of our study was to examine the association between fatigue and problem-solving ability in a sample of Iranian people with MS. Investigation of the linkage between problem-solving ability and fatigue severity could provide preliminary support for making the conceptual premise that addressing such ability might be of importance to people with MS in helping them deal with fatigue.

## Materials and Methods

This cross-sectional, descriptive quantitative study, completed between February 2019 and May 2019, obtained a convenience sample of people with MS who were selected non-randomly from the roster of the Shiraz Multiple Sclerosis Association, Shiraz, Iran. Using a statistical software, the minimum required sample size of 85 subjects was calculated where α = 0.05, β = 0.2, and r = 0.3 (an estimated correlation coefficient). Eligible subjects who met the inclusion criteria were those (a) with a definite diagnosis of MS (all subtypes) made by a neurologist, (b) ages of 18-75 years, (c) able to walk independently, and (d) mentally competent enough to understand and complete research self-reported questionnaires. Provided with a list of potential participants, a research assistant phoned them, and made appointments to visit those who were interested in taking part in the study. She read the questions for those unable to do it on their own. More than 300 people were excluded due to not meeting the inclusion criteria, reluctance to participate, or poor comprehension in the procedures.

To obtain data on two research variables including problem-solving and fatigue, two self-report questionnaires were used. Levels of problem-solving ability was measured with a 24-item standardized Persian version of Cassidy Problem-Solving Inventory (PSI).^7^ For this questionnaire, negative items were recoded, so that a higher value indicated by the questionnaire contributed to the higher levels of the problem-solving construct. Examinees were required to circle ‘Yes’, ‘No’, or ‘Don’t Know’ that corresponded to each questionnaire item. The total score of the scale (range 0-24) was used for the statistical analysis purposes of this study, whereby higher scores indicated higher levels of problem-solving capacity. 

The Fatigue Severity Scale (FSS), a 9-item 7-point Likert scale, was administered to examinees, so that they could best describe their degrees of fatigue severity by choosing a number from 1 to 7 to indicate their degree of agreement with each scale item.^8^ The total scale scores were divided by 9, resulting in the score ranges from 1 to 7, whereby higher scores identified examinees with higher degrees of fatigue. Persian version of FSS exhibited good evidence of psychometric properties for use in people with MS.^9^

The Institutional Review Board at School of Rehabilitation Sciences (Shiraz University of Medical Sciences, Iran), approved this study. Informed consent was obtained from all participants. Using Pearson’s Product Moment Correlation Coefficient, SPSS software (version 23, IBM Corporation, Armonk, NY, USA) was applied to analyze the data. Descriptive statistics were also used to provide estimations of means and standard deviations for research variables. Significance was set at P < 0.050 for the test.

## Results

The mean age of the participants was 38.3 ± 8.9 years, and mean disease duration was 7.5 ± 5.4 years. A statistically significant difference was found for gender distribution (χ^2^ = 49.7, P < 0.001). Namely, women composed 88.2% (n = 75) of the participants. No significant gender difference was found for mean scores of PSI [men (17.5 ± 2.4), women (15.7 ± 3.8), P = 0.052] and FSS [men (4.1 ± 1.3), women (4.1 ± 1.6), P = 0.949]. The mean value for the scores obtained from the PSI and FSS were as follows: PSI [15.9 ± 3.7, range (6-21)], and FSS [4.1 ± 1.6, range (1-7)]. Table 1 shows the main demographic characteristics of the participants.

For Pearson’s Product Moment Correlation Coefficient, preliminary analyses were performed to ensure no violation of the assumptions of normality, linearity, and homoscedasticity. There was a moderate negative correlation between the two variables, namely problem-solving and fatigue, [r = -0.381, P = 0.009], whereby higher levels of problem-solving ability was associated with lower degrees of fatigue.

**Table 1:** Demographic data of participants (n = 85)

**Variables**	**Value**
Age (year) (%)	
≤ 29	14.1
30-49	74.1
≥ 50	11.8
Gender (%)	
Women	88.2
Men	11.8
Educational level (%)	
Up to diploma	67.1
Undergraduate college	28.2
Postgraduate college	4.7
Employment status (%)	
Unemployed	70.6
Employed	20.0
Retired	9.4

## Discussion

This study suggested a moderate, negative association between levels of problem-solving ability and degrees of fatigue in people with MS. Although, the correlation coefficient was moderate, this finding was in line with the previous recent research^5^ that supported the theory of linkage between cognition and fatigue in MS. This finding was consistent with the results of another study, which revealed an association between lower levels of fatigue and higher level of cognitive functioning.^10^ Hence, it could conceivably be assumed that the combination of cognitive deficits and fatigue should be considered by healthcare practitioners, if they want to address patient’s daily life needs.^11^

Our results corroborated the idea that cognitive ability in people with MS might be associated with fatigue severity measured by self-reported fatigue outcome measures.^12^ An implication of this is the possibility that cognitive therapy, aimed at reducing cognitive deficits, might be useful for controlling fatigue in MS. Consequently, studies with more rigorous methodology will be needed to determine which aspect of cognitive capacity, such as memory, problem-solving or attention, merits more consideration for clinical improvement.^13^ For example, following a working memory training strategy, a sample of people with MS experienced significant decrease in their self-reported fatigue symptoms.^12^ Since deficits in problem-solving ability, one aspect of cognition, was reported as a common cognitive impairment in people with MS,^14^ it seems possible to speculate that cognitive training aimed at addressing this deficit may help patient become better at controlling their fatigue, according to finding of this study. Further, with more studies addressing one’s ability to anticipate his/her potential fatigue, monitor current fatigue, and/or prevent exhaustion, the association of problem-solving ability and fatigue can be explored more in depth.

This study experienced certain limitations regarding the sampling procedure and methodology such that this study can only serve as a pilot to design more rigorous research. This descriptive study allows finding a non-causal relationship between problem-solving ability and fatigue severity in MS. If the effectiveness of problem-solving training is to be discovered, future research should include randomized controlled trials.

## Conclusion

In conclusion, the results of our study suggest a moderate, negative correlation between problem-solving and fatigue in MS. It seems that higher levels of problem-solving may be associated with lower degrees of fatigue. Yet, experimental investigations are needed to clarify a causal linkage between the two constructs.

## Notes:

**How to cite this article:** Derakhshanrad SA, Piven E. The relationship between problem-solving ability and fatigue severity in people with multiple sclerosis. Curr J Neurol 2020; 19(1): 36-9.

## References

[1] Thompson AJ, Banwell BL, Barkhof F, Carroll WM, Coetzee T, Comi G (2018). Diagnosis of multiple sclerosis: 2017 revisions of the McDonald criteria. Lancet Neurol.

[2] Goretti B, Portaccio E, Ghezzi A, Lori S, Moiola L, Falautano M (2012). Fatigue and its relationships with cognitive functioning and depression in paediatric multiple sclerosis. Mult Scler.

[3] Chiaravalloti ND, DeLuca J (2008). Cognitive impairment in multiple sclerosis. Lancet Neurol.

[4] Rogers JM, Panegyres PK (2007). Cognitive impairment in multiple sclerosis: Evidence-based analysis and recommendations. J Clin Neurosci.

[5] Grech LB, Kiropoulos LA, Kirby KM, Butler E, Paine M, Hester R (2017). Executive function is an important consideration for coping strategy use in people with multiple sclerosis. J Clin Exp Neuropsychol.

[6] de Rodez Benavent SA, Nygaard GO, Harbo HF, Tonnesen S, Sowa P, Landro NI (2017). Fatigue and cognition: Pupillary responses to problem-solving in early multiple sclerosis patients. Brain Behav.

[7] Cassidy T, Long C (1996). Problem-solving style, stress and psychological illness: Development of a multifactorial measure. Br J Clin Psychol.

[8] Krupp LB, LaRocca NG, Muir-Nash J, Steinberg AD (1989). The fatigue severity scale. Application to patients with multiple sclerosis and systemic lupus erythematosus. Arch Neurol.

[9] Azimian M, Shahvarughi Farahani A, Dadkhah A, Fallahpour M, Karimlu M (2009). Fatigue Severity Scale: The Psychometric properties of the persian-version in patients with multiple sclerosis. Int J Biol Sci.

[10] Kinsinger SW, Lattie E, Mohr DC (2010). Relationship between depression, fatigue, subjective cognitive impairment, and objective neuropsychological functioning in patients with multiple sclerosis. Neuropsychology.

[11] Penner IK (2016). Evaluation of cognition and fatigue in multiple sclerosis: daily practice and future directions. Acta Neurol Scand.

[12] Vogt A, Kappos L, Calabrese P, Stocklin M, Gschwind L, Opwis K (2009). Working memory training in patients with multiple sclerosis - comparison of two different training schedules. Restor Neurol Neurosci.

[13] Mitolo M, Venneri A, Wilkinson ID, Sharrack B (2015). Cognitive rehabilitation in multiple sclerosis: A systematic review. J Neurol Sci.

[14] Beatty WW, Monson N (1996). Problem solving by patients with multiple sclerosis: Comparison of performance on the Wisconsin and California Card Sorting Tests. J Int Neuropsychol Soc.

